# Overshooting Subcellular Redox-Responses in Rett-Mouse Hippocampus during Neurotransmitter Stimulation

**DOI:** 10.3390/cells9122539

**Published:** 2020-11-24

**Authors:** Karina Festerling, Karolina Can, Sebastian Kügler, Michael Müller

**Affiliations:** 1 Zentrum Physiologie und Pathophysiologie, Institut für Neuro- und Sinnesphysiologie, Georg-August-Universität Göttingen, Universitätsmedizin Göttingen, Humboldtallee 23, D-37073 Göttingen, Germany; kafe85@googlemail.com (K.F.); karolina.can@gmail.com (K.C.); 2 Klinik für Neurologie, Georg-August-Universität Göttingen, Universitätsmedizin Göttingen, Robert-Koch Straße 40, D-37075 Göttingen, Germany; sebastian.kuegler@med.uni-goettingen.de

**Keywords:** oxidative stress, reactive oxygen species (ROS), disease progression, Mecp2, hippocampus, roGFP, mitochondria, NADPH oxidase, xanthine oxidase

## Abstract

Rett syndrome (RTT) is a neurodevelopmental disorder associated with disturbed neuronal responsiveness and impaired neuronal network function. Furthermore, mitochondrial alterations and a weakened cellular redox-homeostasis are considered part of the complex pathogenesis. So far, overshooting redox-responses of MeCP2-deficient neurons were observed during oxidant-mediated stress, hypoxia and mitochondrial inhibition. To further clarify the relevance of the fragile redox-balance for the neuronal (dys)function in RTT, we addressed more physiological stimuli and quantified the subcellular redox responses to neurotransmitter-stimulation. The roGFP redox sensor was expressed in either the cytosol or the mitochondrial matrix of cultured mouse hippocampal neurons, and the responses to transient stimulation by glutamate, serotonin, dopamine and norepinephrine were characterized. Each neurotransmitter evoked more intense oxidizing responses in the cytosol of MeCP2-deficient than in wildtype neurons. In the mitochondrial matrix the neurotransmitter-evoked oxidizing changes were more moderate and more uniform among genotypes. This identifies the cytosol as an important reactive oxygen species (ROS) source and as less stably redox buffered. Fura-2 imaging and extracellular Ca^2+^ withdrawal confirmed cytosolic Ca^2+^ transients as a contributing factor of neurotransmitter-induced redox responses and their potentiation in the cytosol of MeCP2-deficient neurons. Chemical uncoupling demonstrated the involvement of mitochondria. Nevertheless, cytosolic NADPH- and xanthine oxidases interact to play the leading role in the neurotransmitter-mediated oxidizing responses. As exaggerated redox-responses were already evident in neonatal MeCP2-deficient neurons, they may contribute remarkably to the altered neuronal network performance and the disturbed neuronal signaling, which are among the hallmarks of RTT.

## 1. Introduction

Rett syndrome (RTT) is a progressive neurodevelopmental disorder, affecting females with a prevalence of about 1:15,000 live births worldwide [1,2]. Stereotypic hand movements, motor dysfunction, microcephaly, rapid regression, loss of speech, cognitive impairment, epilepsy as well as breathing irregularities, are among the main characteristics of RTT. Due to the delayed postnatal manifestation of these symptoms and the initial apparently normal development for up to 18 months after birth, an immediate diagnosis of RTT is often challenging [3,4,5,6]. Nevertheless, even during the initial development first indications of the disease may be evident [7,8].

The primary genetic origins of RTT are spontaneous mutations in the X-linked methyl-CpG-binding protein 2 (*MECP2*) gene, which were confirmed for ~95% of the typical RTT cases [9]. Meanwhile, hundreds of different *MECP2* mutations were identified, which result in a broad severity range of clinical phenotypes and severities [10,11]. Since MeCP2 acts as a transcriptional regulator, directly controlling the activity of various genes, it plays a crucial role in neuronal development, neuronal differentiation and synaptic plasticity [3,12,13,14,15,16]. Although neither gliosis, demyelization nor an obvious neurodegeneration have ever been reported in RTT, analyses of post mortem brain tissue of Rett patients revealed a decreased size of various brain regions such as hippocampus, cortex, thalamus, basal ganglia and amygdala [17,18]. On the cellular level, a reduced dendritic complexity as well as more intensely packed neurons are evident in the cerebral tissue of Rett patients and mouse models of RTT [19,20].

These alterations are accompanied by dysregulated and imbalanced neurotransmitter systems. RTT involves not only lowered levels of dopamine [21,22,23,24,25] and serotonin [26], but also cholinergic deficiencies as well as reduced norepinephrine levels were reported [27,28,29]. In contrast, increased levels of potentially neurotoxic glutamate have been detected in the cerebrospinal fluid of female Rett patients [30,31]. Furthermore, neuropeptides are affected. In the cerebrospinal fluid of RTT patients, the levels of nerve growth factor and substance P were significantly lower than in control subjects [32,33,34,35], whereas endorphin levels were increased [36]. The reduced substance P and serotonin levels, in concert with impaired neurotrophin signaling, might further disrupt the function of the autonomic nervous system, provoking serious breathing irregularities in RTT [37].

Yet, an altered neurotransmission might also arise from changes in neuronal morphology. RTT is considered a synaptopathy with a reduced degree of cellular interaction and a decreased extent of synaptic connectivity among the excitatory (glutamatergic) cortical pyramidal neurons [18,20]. Studies in RTT mouse models further confirmed that pyramidal neurons exhibit fewer dendritic spines as well as decreased spine sizes, thus receiving less excitatory afferents [20]. The logical consequence of such diminished glutamatergic neurotransmission is an excitatory/inhibitory imbalance and a secondary weakening of neurotransmission, leading to hyperexcitability of the neuronal network and promoting the occurrence of seizures [38,39]. Hippocampal hyperexcitability and synaptic instability have been confirmed for mouse models of RTT [40,41]. In contrast, in cortical circuits, inhibition rather than excitation seems to manifest, giving rise to hypoexcitability [42].

Detailed studies also indicate a pivotal role for mitochondria in plasticity-related mechanisms such as control of neurite outgrowth or neuronal polarity [43,44,45,46]. Since functionally impaired mitochondria may markedly contribute to reactive oxygen species (ROS) formation, they can also be among the causes of altered cellular signaling and disturbed neuronal plasticity [47,48]. Impaired mitochondrial morphology, for example, spherical mitochondria, low-density structure of mitochondrial matrix, rudimentary cristae and abundant ribosomal content, granular matrix inclusions and disorganized membranous material, have been identified in Rett patients [49,50,51]. More importantly, the function and metabolism of mitochondria is also affected in RTT. Data from muscle and frontal cortex biopsies of RTT patients revealed lowered levels of cytochrome *c* oxidase and succinate cytochrome *c* reductase together with a proton leak across the inner mitochondrial membrane [52,53,54]. In vitro analyses on MeCP2-deficient hippocampal neurons confirmed a more vulnerable cellular redox balance under baseline control conditions as well as exaggerated redox responses to either acute oxidative challenge, mitochondrial inhibition, or both [55,56]. This redox-imbalance does not only affect neurons but also glial cells [57]. Mechanistically, the redox-imbalance in RTT arises from a combination of increased mitochondrial ROS release, intensified extramitochondrial ROS generation as well as a less efficient cell-endogenous detoxification of oxidants [58,59,60,61].

Among the characteristics of RTT is that it is not associated with a marked neurodegeneration [62], but rather with a miscommunication of neuronal elements, disturbed synaptic functions and alterations in neuronal excitability [3,63]. To determine to what extent neuronal communication and interactions may challenge the fragile cellular redox-balance and redox-homeostasis, we characterized the (sub)cellular redox-responses resulting from specific neurotransmitter stimulation of cultured hippocampal neurons and defined the respective alterations in *Mecp2*^−/*y*^ mice as compared to WT mice.

## 2. Material and Methods

### 2.1. Mouse Model for RTT

The “Bird strain” mouse model (*Mecp2^tm1.1Bird^*) was used, which lacks exons 3 and 4 of the *Mecp2* gene and shows a complete loss of function of MeCP2 [64]. MeCP2-deficient male mice (*Mecp*^−/*y*^) develop the characteristic phenotype with hindlimb clasping, growth arrest, breathing irregularities and premature mortality beyond postnatal day 50. The heterozygous female mice of this disease model (*Mecp2*^+/−^) exhibit a cellular mosaicism and hence a more variable, less severe phenotype with markedly delayed disease onset [64]. For the sake of controlled and uniform conditions, in other words, the total lack of MeCP2, our cell-based study was therefore performed on male WT and *Mecp2*^−/*y*^ mice. Breeding of these mice as well as all in vitro experiments and procedures were in accordance to German national regulations as well as European guidelines for animal welfare, and were approved by the Office of Animal Welfare of the University Medical Center Göttingen as well as by the Lower Saxony State Office for Consumer Protection and Food Safety (file number G16/2177). Foster mice (NMRI strain) were used for maternal care, to improve the stability of our mouse colony.

### 2.2. Solutions

The majority of chemicals were obtained from Sigma-Aldrich (St. Louis, MO, USA), other vendors are clearly stated. The artificial cerebrospinal fluid (ACSF) served to maintain cellular viability during the optical recordings. It was made of 130 mM NaCl, 3.5 mM KCl, 1.25 mM NaH_2_PO_4_, 24 mM NaHCO_3_, 1.2 mM CaCl_2_, 1.2 mM MgSO_4_ as well as 10 mM dextrose, and a pH of 7.4 was maintained by constant aeration with 95% O_2_ plus 5% CO_2_ (carbogen). For nominally Ca^2+^-free solutions, CaCl_2_ was omitted from the ACSF. Norepinephrine, serotonin, dopamine and allopurinol were added directly to the ACSF in their desired concentrations immediately before use. This is crucial in particular for dopamine and norepinephrine, as these compounds autoxidize easily and may generate extracellular H_2_O_2_ [65]. Glutamate (sodium salt) was prepared as aqueous stock solutions of 5 mM, and kept frozen (−20 °C). Carbonyl cyanide-4(trifluoromethoxy) phenylhydrazone (FCCP, Tocris Bioscience, Bristol, UK) and diphenyleneiodonium chloride (DPI, Tocris Bioscience) were dissolved as 10 mM stocks in dimethyl sulfoxide (DMSO) and stored at −20 °C. The Ca^2+^-sensitive dye Fura-2 AM (ThermoFisher Scientific, Waltham, MA, USA) was dissolved as 1 mM stock solution in DMSO containing 10% Pluronic F-127 and stored at −20 °C.

### 2.3. Dissociated Cell Cultures

Hippocampal cell cultures were prepared from *Mecp2^−/y^* and WT males at postnatal day 2–5 as described in detail earlier [57]. Upon isolation, the brain was submerged in ice-cold Hank’s balanced salt solution (HBSS) containing 20% fetal calf serum (FCS). Hippocampi were isolated and divided into 8–10 pieces, which were then washed repeatedly in HBSS to remove the remaining FCS. After trypsin-digestion in the incubator (10 min, 37 °C, 5% CO_2_), washing steps first with HBSS and then with HBSS including 20% FCS were conducted. Hippocampi were then triturated in a dissociation solution and centrifuged (1500 rpm, 10 min, 4 °C). The isolated pellet was re-dissolved in plating medium. About 30,000–40,000 cells were plated on each of the Matrigel (BD Biosciences, Franklin Lakes, NJ, USA)-coated glass coverslips (Nunc, 13 mm diameter), and maintained in 4-well plates (Nunc) in the incubator (37 °C, humidified atmosphere, 5% CO_2_). Depending on brain size, 12–16 coverslips could be prepared from the hippocampi of a single mouse. After 24 h the plating medium was replaced by growth medium, which was then partially (50%) refreshed every 2–3 days during the culturing period. The experiments were performed between 7 and 16 days in vitro (DIV).

### 2.4. Culturing Media

All culturing media were sterile filtered and then stored at 4 °C. HBSS consisted of Hanks’ balanced salts, 0.35 g/L NaHCO_3_, and 0.238 g/L HEPES; the pH was adjusted with 1 M NaOH to 7.3–7.4. Digestion solution contained 137 mM NaCl, 5 mM KCl, 7 mM Na_2_HPO_4_ and 25 mM HEPES. Right before use, 0.5 g/100 mL trypsin and 0.05 g/100 mL DNAse were added, and the pH was adjusted to 7.2. The dissociation solution was made of Hanks’ balanced salts, 12 mM MgSO_4_ and 0.05 g/100 mL DNAse. Plating medium consisted of minimum essential medium (MEM) (ThermoFisher Scientific, Waltham, MA, USA), 10% FCS, 5 mg/mL glucose, 0.2 mg/mL NaHCO_3_, 0.1 mg/mL transferrin (Calbiochem, San Diego, CA, USA), 2 mM L-glutamine, and 25 µg/mL insulin. Growth medium contained MEM, 5 mg/mL glucose, 0.2 mg/mL NaHCO_3_, 0.1 mg/mL transferrin, 5% FCS, 0.5 mM L-glutamine, 20 µL/mL B27 50× supplement including antioxidants (Invitrogen, Carlsbad, CA, USA), 2 µM cytosine arabinoside, and 100 µg/mL penicillin-streptomycin (Biochrom, Cambridge, UK).

### 2.5. roGFP Transduction

The genetically-encoded optical redox sensor reduction oxidation-sensitive green fluorescent protein 1 (roGFP1) [66] was expressed under the control of the synapsin-1 promoter in either the mitochondrial matrix (roGFPm) or the cytosol (roGFPc) of neurons, by taking advantage of the earlier developed AAV-6 constructs. Including the mitochondrial targeting sequence of subunit VIII of cytochrome C oxidase ensured the expression of roGFP in the mitochondrial matrix, without any further targeting sequences roGFP was expressed in cytosol [55,67]. Transduction was performed on DIV 2, by adding 2.5 µL of diluted virus constructs (stock dilution 1:50, stored in phosphate buffered saline (PBS) at −80 °C) into 800 µL of growing medium. Obvious differences in the transduction efficiency and the cellular viability/survival could not be observed among WT and *Mecp2*^−/*y*^ cultures.

### 2.6. Immunostaining

MitoTracker RED FM (Life Technologies, Carlsbad, CA, USA) served to label mitochondria. Cultures were incubated for 20 min (1 μM, 37 °C, 5% CO_2_). To visualize mitochondria in vitro, a 2-photon microscope with a 63× 1.0NA objective (Plan-Apochromat VIS-IR, Zeiss, Oberkochen, Germany), and an excitation wavelength of 860 nm was used [57].

The neuron specific expression of roGFP was confirmed by immunostaining with the neuronal marker anti-microtubule associated protein 2 (MAP2) antibody (cell signaling). After two washing cycles (3 min each) with pre-warmed PBS (37 °C), the neuronal cultures were fixed in PBS with 4% paraformaldehyde (PFA) for ~20 min at room temperature. For permeabilization, cells were exposed twice to 0.2% triton in PBS (3 min each treatment) and incubated overnight with the MAP2 antibody (stock dilution 1:300, 4 °C). Neuronal cultures were washed twice with 0.2% triton in PBS for 3 min and the secondary antibody (anti-rabbit Cy3, Sigma-Aldrich, St. Louis, MO, USA) was added for 1 h at room temperature. After double washing with 0.2% triton in PBS (3 min each) and the final two times washing in PBS, neurons were dried and mounted (DakoCytomation, Glostrup, Denmark) on glass microscope slides. To visualize neurons expressing MAP2, a fluorescence microscope (BX51WI, Olympus, Shinjuku, Japan) with a 60×/0.90NA objective (Olympus LUMPlanFI) and an excitation wavelength of 525 nm were used.

### 2.7. Optical Recordings and 2-Photon Imaging

Subcellular redox conditions were monitored with the redox sensor roGFP1 [66], following our earlier established calibration routines and CCD-camera based imaging procedures [55,67,68]. The roGFP indicator is ratiometric by excitation, as its two discrete absorption peaks respond oppositely to oxidation and reduction [66]. Therefore, we excited roGFP alternately at 395 and 470 nm (0.1 Hz frame rate, 4 × 4 pixel binning) and calculated the resulting fluorescence ratio R_395nm/470nm_. Dynamics of subcellular redox changes were visualized using a fast-switchable light source (Polychrome II; Till Photonics, Gräfelfing, Germany) and a sensitive CCD-camera (Imago QE; PCO Imaging, Kelheim, Germany), both of which were controlled by the TILLvisION device-control software package (version 4.0.1; TILL Photonics, Gräfelfing, Germany), and a 60× 0.9NA water immersion objective (LUMPlan FI, Olympus, Shinjuku, Japan).

To image hippocampal cultures expressing roGFPc and roGFPm at higher resolution, a 2-photon laser scanning microscope (TriMScope II with an upright Olympus BX51WI microscope, LaVision BioTec, Bielefeld, Germany) was used [57]. The roGFP sensors were excited at a wavelength of 890 nm and a 63 × 1.0 NA objective (Plan-Apochromat VIS-IR, Zeiss, Oberkochen, Germany) was used.

Cytosolic Ca^2+^ changes were monitored using the Ca^2+^-specific dye Fura-2 AM. Dye-loading of the cell-cultures was performed in the incubator (30 min, 37 °C, 5% CO_2_) by adding 5 µM Fura-2 directly to the growing medium. Real-time imaging of cytosolic Ca^2+^ transients was performed with the above mentioned Till Photonics CCD-camera imaging system and a 63 × 1.0 NA objective (Plan-Apochromat VIS-IR, Zeiss, Oberkochen, Germany). Fura-2 was excited alternately at 345 nm and 380 nm, the resulting fluorescence emissions recorded at 510 nm, and the fluorescence ratio F345/F380 was calculated. A frame rate of 0.1 Hz together with 4 × 4 pixel binning was applied. All imaging experiments were run in a submersion style chamber (volume 2.5 mL), and to allow for stable optical recordings, the superfusion flow rate was limited to 4 mL/min. To ensure a rapid onset of the transmitter-mediated effects, once the compounds reached the chamber, relatively high transmitter concentrations were chosen.

### 2.8. Calibration of roGFP

Quantitative redox imaging demands the proper calibration of the roGFP response range in the respective cell compartments. Therefore, roGFP was forced into full oxidation (5 mM H_2_O_2_, 5 min) and full reduction (10 mM DTT, 5 min) to obtain the respective ratiometric values (R_ox_, R_red_). The instrument factor F470_ox_/F470_red_ represents the fluorescence intensities at 470 nm excitation observed during maximum oxidation and reduction [69]. The standard redox potential of the roGFP1 variant of this sensor we used here (E^0′^_roGFP_) is −291 mV [66]. Based on these parameters, the relative degrees of roGFP oxidation (OxD_roGFP_) and the corresponding roGFP redox potentials (E_roGFP_) were then calculated for the roGFP fluorescence ratios (R) recorded in the cytosol and the mitochondrial matrix:

(1) $${\mathrm{OxD}}_{\mathrm{roGFP}}=\frac{R-R_{\mathrm{red}}}{\frac{F\;470\mathrm{ox}}{F\;470\mathrm{red}}\;\left(R_{\mathrm{ox}}-R\right)+\left(R-R_{\mathrm{red}}\right)}$$

(2) $$E_{\mathrm{roGFP}}={E^{0}{}^{\prime }}_{\mathrm{roGFP}}-\frac{\mathrm{RT}}{2F}\ln\left(\frac{1-{\mathrm{OxD}}_{\mathrm{roGFP}}}{{\mathrm{OxD}}_{\mathrm{roGFP}}}\right)$$

### 2.9. Calibration of Fura-2

The quantitative Ca^2+^ imaging also requires proper calibrations. Therefore, the Ca^2+^-sensitive fluorescent dye Fura-2 was calibrated in vitro [70] to determine the calibration constants R_min_, R_max_ and the instrument factor F380_max_/F380_min_ (Sf2/Sb2). The calibration solutions contained 140 mM KCl, 1 mM MgCl_2_, 10 mM HEPES as well as 100 μM Fura-2, and they were adjusted to either 0 M Ca^2+^, 300 nM Ca^2+^ or 10 mM Ca^2+^ using BAPTA (1,2-bis(*o*-aminophenoxy)ethane-*N*,*N*,*N′*,*N′*-tetraacetic acid) [70]. The determined dissociation constant K_d_ of 225 nM matches earlier reports [71]. Based on these calibration parameters, the Fura-2 ratios were then converted to cytosolic Ca^2+^ concentrations:

(3) $$\left({\mathrm{Ca}}^{2+}\right)=K_{d}\times \frac{R-R_{\min}}{R_{\max}-R}\times \frac{\mathrm{Sf}2}{\mathrm{Sb}2}$$

### 2.10. Spectrofluorometric Quantification of H_2_O_2_

The extracellular formation of H_2_O_2_, which may arise as a consequence of either norepinephrine, dopamine autoxidation, or both, was quantified in a cuvette-based assay with horseradish peroxidase and Amplex UltraRed (Life Technologies, Carlsbadl, CA, USA; for details see [72]). Formation of H_2_O_2_ is indicated by a stoichiometric accumulation of the oxidized and highly fluorescent Amplex UltroxRed, which was followed in a spectrofluorometer (flx-Xenius, ESV 5.98, SAFAS Monaco, Monaco). Based on parallel calibration experiments with defined H_2_O_2_ standards, Amplex UltroxRed fluorescence intensities were then converted into H_2_O_2_ concentrations [72].

### 2.11. Statistics

The current study was performed on 28 WT and 18 *Mecp2^−/y^* mice. For the various experiments, we made sure that at least two different mice per genotype (range 2–7 WT mice and 2–4 *Mecp2^−/y^* mice) entered each experimental paradigm. For pairwise comparisons, such as genotype-related differences, two-tailed unpaired Student’s *t*-tests were used, given that the data showed a normal distribution (Kolmogorov–Smirnov test). Otherwise, Mann–Whitney rank sum tests were conducted. Multiple group comparisons were done by one-way ANOVA, followed by Holm–Sidak tests. Statistically significant changes are indicated by asterisks (* *p* < 0.05; ** *p* < 0.01; *** *p* < 0.001). Statistical calculations were performed with SigmaStat 3.5 (Systat Software, Erkrath, Germany). All numerical values are given as mean ± standard deviation (SD), and the number of cells (n) is reported.

## 3. Results

### 3.1. Cellular Targeting and Subcellular Expression of roGFP

**Figure 1:**
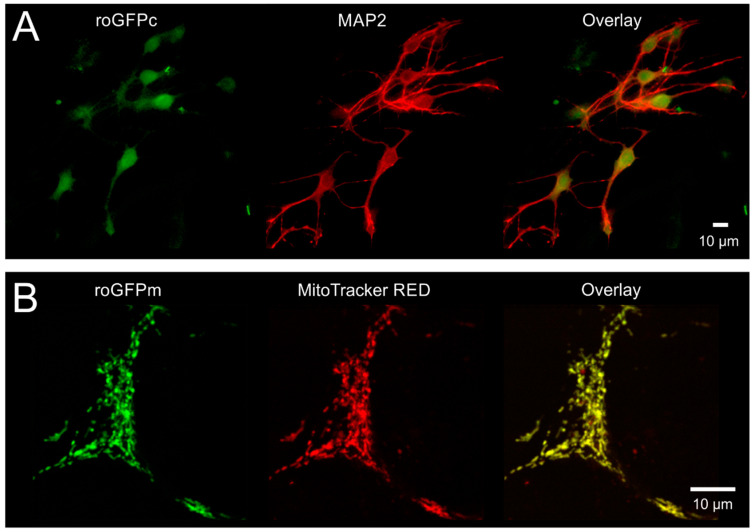
Confirming cellular specificity and proper subcellular targeting of roGFP. (**A**) As proven by MAP2 immunolabeling, roGFPc was expressed homogenously in the cultured hippocampal neurons. Displayed cells were PFA-fixed, immunolabeled and viewed with an epifluorescence microscope. MAP2 and roGFP fluorescence are displayed in pseudo-colors. (**B**) MitoTracker RED FM counterlabeling verified that roGFPm is specifically expressed in the mitochondrial matrix of neurons. MitoTracker-labeling was performed in viable cultures and neuronal mitochondria were visualized with a 2-photon microscope. MitoTracker and roGFP fluorescence are shown in pseudo-colors.

To confirm the neuron-specific expression of roGFP and its proper subcellular targeting, we fixed (4% PFA) and co-labeled hippocampal neuronal cultures with MAP2-directed antibodies. As expected, roGFPc fluorescence fully matched the MAP2-labeling (Figure 1A). The mitochondria-specific expression of roGFPm was verified by co-labeling viable cell cultures with MitoTracker RED FM. Again, a perfect colocalization was confirmed (Figure 1B).

### 3.2. Response Calibrations of roGFPc and roGFPm

**Figure 2:**
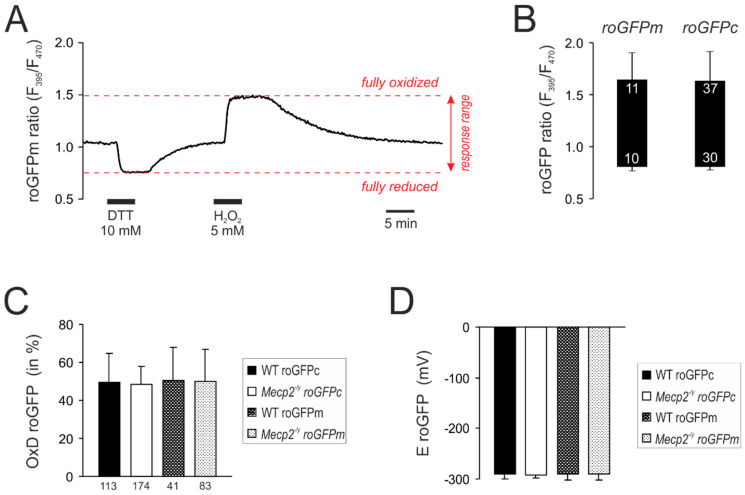
Calibration of the response ranges of roGFPc and roGFPm. (**A**) Calibration experiment performed for roGFPm. Reduction of roGFPm decreases the fluorescence ratio (F395/F470), whereas oxidation corresponds to an increased ratio. Maximum reduction and oxidation of roGFPm was evoked by DTT and H_2_O_2_, respectively. Ideally, both treatments are to be recorded in the same cell, also alternating the order of treatments among cells. (**B**) Box plot of the determined responses ranges. The ceiling of each box identifies the maximally oxidized roGFP ratio; the floor indicates the maximally reduced state. Error bars represent standard deviations of the ratiometric values. Response ranges were identical for cytosol-expressed (roGFPc) and mitochondria-targeted roGFP (roGFPm). Numerical figures in the box indicate how many calibrations were performed. They slightly differ for full oxidation and reduction, as not each cell undergoing maximum oxidation as a first treatment fully recovered and therefore did not undergo the following reducing treatment. (**C**) Steady state resting baseline conditions determined in cytosol and mitochondrial matrix of WT and *Mecp2^−/y^* neurons. Plotted is the relative degree of roGFP oxidation, as calculated from the calibration parameters (see Equation (1)). The number of cells analyzed is indicated below each bar. (**D**) Summary of the roGFP reduction potentials calculated for the different cell compartments and genotypes (see Equation (2)).

For quantitative redox analyses, the roGFP response ranges were determined in cytosol as well as mitochondrial matrices by treatment with H_2_O_2_ (5 M) and DTT (10 mM, 5 min each), which forced roGFPc and roGFPm into the fully oxidized and the fully reduced state, respectively (Figure 2A). As expected, identical roGFP response ranges were obtained for both cell compartments (Figure 2B). Based on these calibrations, the baseline resting redox conditions were quantified. In WT neurons, roGFPc showed a relative degree of oxidation (OxD_roGFP_) of 49.5 ± 15.2% (*n* = 113), which corresponds to a cytosolic redox potential (E_roGFP_) of −291.0 ± 9.6 mV. Very similar values were obtained for mitochondrial matrices as well as the respective cell compartments in *Mecp2^−/y^* neurons (Figure 2C,D).

### 3.3. Neurotransmitter-Mediated Redox Changes

Previously we confirmed an oxidative burden with intensified responses of *Mecp2^−/y^* hippocampal neurons and glial cells to acute redox challenges such as mitochondrial inhibition, direct oxidant-challenge and severe hypoxia [55,56,57]. Now we assessed whether such overshooting redox-responses occur in the cytosol or the mitochondrial matrix of *Mecp2^−/y^* neurons in response to more physiological events, such as stimulation by the neurotransmitters glutamate, norepinephrine, serotonin and dopamine. Transmitter concentrations were based on earlier reports [73,74,75,76] and if necessary adjusted to levels inducing reproducible redox responses in our specific submersion style chamber (2.5 mL volume, 4 mL/min flow rate).

**Figure 3:**
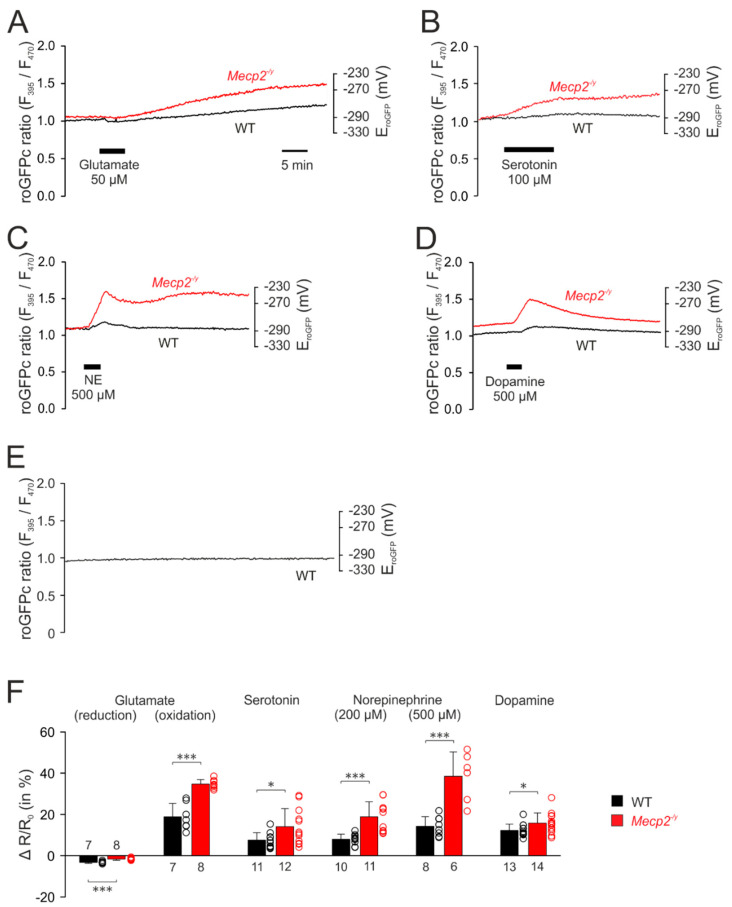
*Mecp2^−/y^* neurons show intensified cytosolic redox-responses to neurotransmitter stimulation. (**A**) Glutamate induced an initial reducing shift. It then turned into a clear oxidation of roGFPc, which was markedly more intense in *Mecp2^−/y^* neurons. Plotted are the roGFP ratios and on the right-hand ordinate the corresponding redox potentials are indicated. Time scaling also applies to all other panels. (**B**) Serotonin also mediated a more intense oxidation of roGFPc in *Mecp2^−/y^* neurons. (**C**) Norepinephrine shifted roGFPc fluorescence to a more oxidized level in *Mecp2^−/y^* neurons. (**D**) Dopamine induced fully reversible oxidizing shifts in both genotypes, which again were more pronounced in *Mecp2^−/y^* than in WT neurons. (**E**) No changes in roGFPc fluorescence were evident, when neurons were just imaged for ~60 min without any additional treatment (*n* = 6). (**F**) Statistical summary of the neurotransmitter-mediated redox-alterations in WT and *Mecp2^−/y^* neurons. Plotted are the normalized transmitter-induced changes in the roGFPc fluorescence ratio (ΔR/Ro). On the right-hand of each bar, the scatter of the respective data is indicated as a dot plot. Genotype-related differences are indicated by asterisks (* *p* < 0.05, *** *p* < 0.001; unpaired *t*-test/Mann–Whitney rank sum test). The number of cells tested for a given treatment is reported for each bar.

The first set of experiments addressed the cytosolic redox homeostasis. Application of glutamate (50 µM, 5 min) induced biphasic responses in cytosolic redox balance, consisting of an initial decrease, in other words, a reducing shift in the roGFPc ratio, followed by a secondary continuous increase, in other words, an oxidation (Figure 3A). Since the roGFPc ratio failed to recover to its pre-treatment baseline after wash-out of glutamate, the redox-level reached 30 min after the end of glutamate application was used for quantification and genotypic comparison; for the other neurotransmitters the respective peak values of their redox responses were analyzed. In the case of glutamate stimulation, the initial reduction of the roGFPc ratio was significantly more intense in WT (−3.3 ± 0.5%, *n* = 7) than in *Mecp2^−/y^* neurons (−1.7 ± 0.5%; *n* = 8). Yet, the secondary oxidation was markedly more intense in *Mecp2^−/y^* (34.6 ± 2.2%; *n* = 8) than in WT neurons (18.8 ± 6.4%, *n* = 7) (Figure 3F).

Serotonin (100 µM, 10 min) also shifted the roGFPc ratio towards oxidation, which was clearly more pronounced in *Mecp2^−/y^* (14.0 ± 8.8%; *n* = 12) than in WT neurons (7.5 ± 3.6%; *n* = 11; Figure 3B,F).

Norepinephrine, applied in two different doses (200 µM and 500 µM, 3 min each), evoked an increase in the roGFPc ratio, which was followed by a partial recovery after wash-out and then a maintained longer lasting oxidation of the roGFPc ratio or even a slow secondary further increase (Figure 3C). Similarly, *Mecp2^−/y^* neurons showed more intense oxidizing responses of 18.9 ± 7.2% (*n* = 11) as compared to WT neurons (7.9 ± 2.5%, *n* = 10). In response to 500 µM norepinephrine the roGFPc ratio rose by 38.5 ± 11.8% (*n* = 6) in *Mecp2^−/y^* and by 14.2 ± 4.7% in WT neurons (*n* = 8) (Figure 3F).

Dopamine (500 µM, 3 min) induced oxidizing responses in the roGFPc ratio, which—upon washout—fully recovered (Figure 3D). In detail, the roGFPc ratio increased by 15.7 ± 5.0% in *Mecp2^−/y^* neurons (*n* = 14), which again was more intense than in WT neurons (12.3 ± 3.0%; *n* = 13) (Figure 3F).

To rule out any cell impairment, sensor bleaching or other effects that may arise from the imaging conditions, we performed control recordings, in which cytosolic redox conditions were monitored continuously with the very exposure times and frame rates for 1 h. These tests clearly confirmed no drift, no bleaching and no continuous increases in redox balance, which rules out any artifacts, impairment of cell viability, or both, by the repeated ratiometric excitation (*n* = 8) (Figure 3E).

Furthermore, as in particular dopamine and norepinephrine become oxidized easily in solutions and give rise to extracellular H_2_O_2_ formation—which by itself would be sufficient to induce redox alterations in the cultured cells—we quantified the H_2_O_2_ formation in a cuvette assay using Amplex UltraRed (see [72]). In the case of ACSF (*n* = 3), 50 µM glutamate (*n* = 6) and 100 µM serotonin (*n* = 6), the H_2_O_2_ content was below the assay’s detection limit of 20 nM. Norepinephrine (500 µM) and dopamine (500 µM) gave rise to only moderate H_2_O_2_ generation. After 5 min, in other words, already beyond the application time of both drugs, H_2_O_2_ levels of the neurotransmitter-containing solutions measured 85.6 ± 37.9 nM (*n* = 4) and 105.9 ± 40.6 nM (*n* = 4), and after 10 min they averaged 422.4 ± 33.8 nM (*n* = 4) and 431.4 ± 34.2 nM (*n* = 4), respectively. None of which is sufficient to induce noticeable cytosolic redox alterations in cultured hippocampal neurons [68].

After observing clearly intensified redox responses to neurotransmitter stimulation in the cytosol of *Mecp2^−/y^* hippocampal neurons, we assessed whether overshooting oxidizing shifts would also be detectable in the mitochondrial matrix. Exposing roGFPm expressing hippocampal cell cultures to the identical neurotransmitter stimulation paradigms revealed that the redox responses in the roGFPm ratio quantified for WT and *Mecp2^−/y^* neurons were more moderate than in the cytosol. Furthermore, the mitochondrial redox changes recovered more readily.

**Figure 4:**
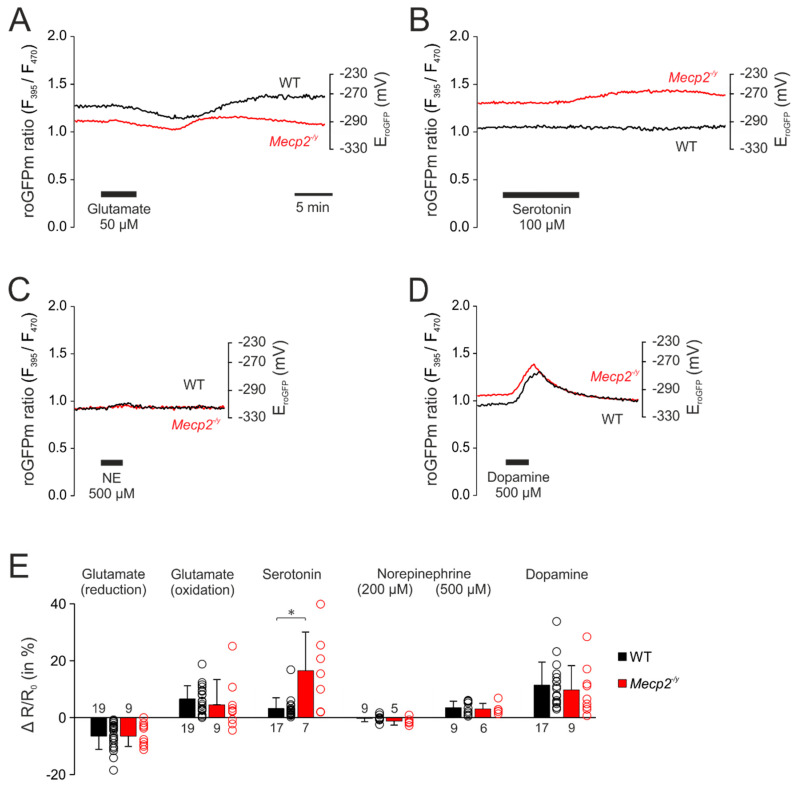
Neurotransmitter stimulation mediated only moderate redox-responses in mitochondrial matrices. (**A**) Glutamate also induced biphasic redox-responses in the mitochondria. The initial reducing shift and the secondary oxidation of roGFPm did, however, not differ among genotypes. (**B**) Serotonin mediated a moderate oxidation of roGFPm, which was clearly more intense in *Mecp2^−/y^* than in WT neurons. (**C**) Norepinephrine evoked only a very moderate oxidation of roGFPm, which was identical in both genotypes. (**D**) Dopamine mediated comparable oxidizing shifts in the mitochondrial matrix of WT and *Mecp2^−/y^* neurons. (**E**) Summarizing bar plot of neurotransmitter-evoked changes in mitochondrial redox balance. (* *p* < 0.05; Mann–Whitney rank sum test). Plotted are the normalized changes in the roGFP ratio (ΔR/Ro), and the dot-plots signify the scatter of the underlying data.

Glutamate (50 µM, 5 min) also induced (in most cases) a biphasic response in the mitochondrial matrix (Figure 4A). The initial reducing shift averaged in WT neurons −6.5 ± 4.7% (*n* = 19) and was similar in *Mecp2^−/y^* neurons (−6.5 ± 3.6%; *n* = 9). It was followed by a secondary oxidizing shift in WT (6.5 ± 4.7%, *n* = 19) and *Mecp2^−/y^* neurons (4.5 ± 8.9%, *n* = 9) (Figure 4A,E). Some of the neurons responded—as also seen in cytosol—with a continuous oxidation (WT n = 3; *Mecp2^−/y^ n* = 3).

Serotonin (100 µM, 10 min) was the only neurotransmitter, which evoked more intense oxidizing responses in *Mecp2^−/y^* neurons (Figure 4B). The roGFPm ratio increased by 16.5 ± 13.6% (*n* = 7) in *Mecp2^−/y^* neurons, but only by 3.2 ± 3.8% in WT cells (*n* = 17, Figure 4E).

In contrast, norepinephrine evoked only very moderate responses. Applied at the lower concentration (200 µM, 3 min) it hardly affected the redox-baseline (Figure 4E). Administration of 500 µM norepinephrine (3 min) slightly increased the roGFPm ratio to a similar degree in WT and *Mecp2^−/y^* neurons (3.5 ± 2.3%, *n* = 9, and 3.1 ± 2.0%, *n* = 6) (Figure 4C,E).

Dopamine (500 µM, 3 min) also evoked fully reversible responses in the mitochondrial matrix. In both, WT (*n* = 17) and *Mecp2^−/y^* neurons (*n* = 9), the roGFPm ratio increased by 11.4 ± 8.1% and 10.8 ± 8.6%, respectively (Figure 4D,E).

### 3.4. Effect of Neurotransmitters on Cytosolic Calcium Influx

A pivotal cellular parameter, which is affected by various neurotransmitters and may provoke the generation of ROS, is the intracellular Ca^2+^ level ((Ca^2+^)_i_). Therefore, we quantified the cytosolic Ca^2+^ transients induced by the different neurotransmitters, using Fura-2 imaging. Due to the spectral overlap of roGFP- and Fura-2-emission, redox- and Ca^2+^-changes could not be monitored simultaneously. Therefore, to assess a potential correlation of the two variables, identical experimental protocols were applied in the Ca^2+^ imaging experiments.

**Figure 5:**
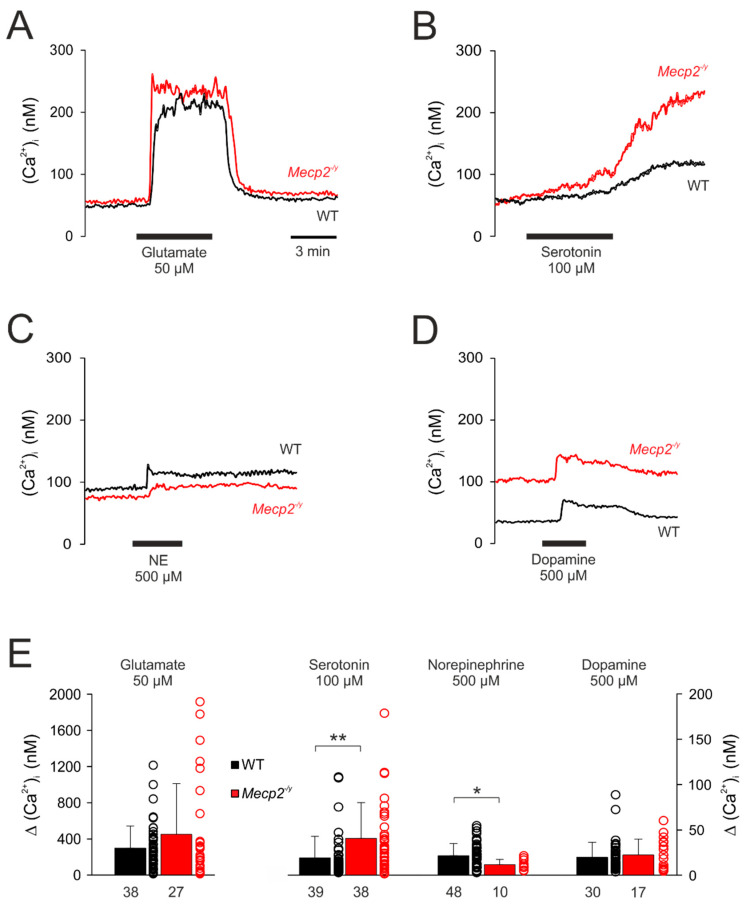
Neurotransmitter-induced cytosolic Ca^2+^ transients. (**A**) Glutamate mediated a sudden, marked increase in (Ca^2+^)_i_ suggesting influx from extracellular space. (**B**) In response to serotonin, the (Ca^2+^)_i_ only increased slowly and then showed a secondary increase, which did not recover within the observation time of ~15 min. (**C**) Norepinephrine induced only moderate changes in (Ca^2+^)_i_, which were slightly more intense in WT neurons and recovered only slowly to baseline conditions. (**D**) Dopamine mediated moderate increases in (Ca^2+^)_i_, which fully recovered and did not differ among genotypes. (**E**) Summary of cytosolic Ca^2+^ transients evoked by neurotransmitter stimulation in WT and *Mecp2^−/y^* neurons. Plotted are the averaged changes in (Ca^2+^)_i_ as well as the scatter range of the underlying data. The number of neurons that underwent a respective treatment is indicated below each bar. Asterisks mark significant differences among WT and *Mecp2^−/y^* neurons (* *p* < 0.05, ** *p*< 0.01; Mann–Whitney rank sum test).

Under resting conditions, the (Ca^2+^)_i_ baseline averaged in WT neurons 67.2 ± 44.9 nM (*n* = 111) and it was slightly higher in *Mecp2^−/y^* neurons (86.2 ± 61.8 nM, n = 50). As expected, glutamate (50 μM, 5 min) evoked a rapid and intense Ca^2+^ rise, which measured 298.1 ± 245.2 nM (*n* = 38) in WT and tended to be slightly higher (451.5 ± 560.9 nM; *n* = 27) in *Mecp2^−/y^* neurons (*p* = 0.08, Figure 5A,E). Upon wash-out, the cytosolic Ca^2+^ levels readily recovered to their baseline levels.

Serotonin (100 µM, 10 min) only evoked a slow increase in (Ca^2+^)_i_, which was slightly more intense in *Mecp2^−/y^* (Δ(Ca^2+^)_i_ (40.8 ± 39.4 nM, n = 38, *p* <0.01)) than in WT neurons (Δ(Ca^2+^)_i_ 19.1 ± 23.8 nM; *n* = 39). After about 10 min the initial moderate rise then accelerated into a secondary and sustained Ca^2+^ increase (Figure 5B,E).

Norepinephrine (500 µM, 3 min) evoked only moderate rises in (Ca^2+^)_i_, which were slightly more intense in WT (21.4 ± 13.5 mM, *n* = 48) than in *Mecp2^−/y^* neurons (11.7 ± 5.7 nM, *n* = 10) and recovered only slowly (Figure 5C,E).

Dopamine (500 µM, 3 min) also elicited only moderate increases in (Ca^2+^)_i_. They fully recovered to baseline conditions and did not differ among genotypes (WT: 19.7 ± 16.5 nM, *n* = 30; *Mecp2^−/y^*: 22.3 ± 17.5 nM, *n* = 17) (Figure 5D,E).

### 3.5. Uncovering the Redox-Signaling Pathways Involved

Since *Mecp2^−/y^* hippocampal neurons showed intensified oxidizing responses to neurotransmitter stimulation, we next elucidated potential cellular mechanisms that may contribute to these exaggerated redox responses. Since the cytosol constitutes the very cellular compartment where most cellular signalling cascades are located and integrated, we monitored cytosolic redox-balance and focused on potential mechanisms such as intracellular Ca^2+^ signaling, mitochondrial function, as well as cytosolic enzymes like NADPH oxidase (NOX) and xanthine oxidase (XO), which are known to be involved in cell-endogenous ROS production [77]. In these experiments, we focused on glutamate and dopamine, because glutamate evoked the most intense and largely irreversible changes in cellular redox-balance, whereas dopamine mediated clear but fully reversible oxidizing responses in the cytosol and the mitochondrial matrix.

First, the dopamine-evoked (500 µM, 3 min) redox responses were characterized upon modulation of potentially contributing cellular ROS sources and then compared among WT and *Mecp2^−/y^* neurons. The respective treatments started 8–10 min prior to neurotransmitter application.

**Figure 6:**
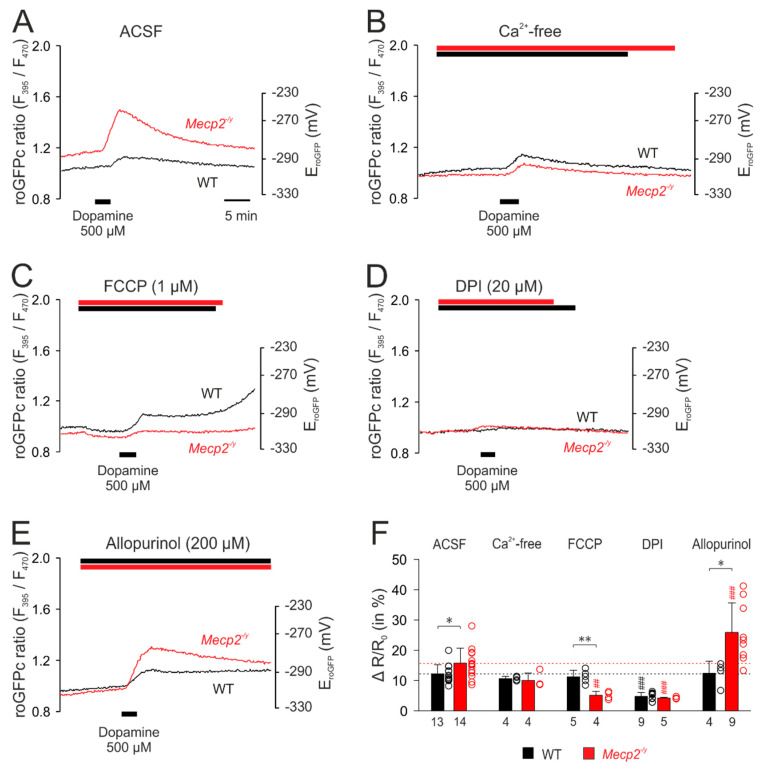
Mitochondria as well as extra-mitochondrial reactive oxygen species (ROS) sources contribute to the dopamine-mediated cytosolic oxidation. (**A**) Dopamine-mediated roGFPc responses observed in WT and *Mecp2^−/y^* neurons under artificial cerebrospinal fluid (ACSF) control conditions. (**B**) Withdrawal of extracellular Ca^2+^ dampened the dopamine-mediated oxidation in *Mecp2^−/y^* neurons only. (**C**) Mitochondrial uncoupling by carbonyl cyanide 4-(trifluoromethoxy)phenylhydrazone (FCCP) moderately reduced roGFPc. Subsequent addition of dopamine evoked a less intense roGFPc oxidation in *Mecp2^−/y^* neurons, whereas the WT responses remained unchanged. (**D**) Inhibition of NADPH oxidase by diphenyleneiodonium chloride (DPI) markedly depressed the dopamine-induced cytosolic oxidation in both, WT and *Mecp2^−/y^* neurons. (**E**) Blocking xanthine oxidase by allopurinol did not modify the responses of WT neurons to dopamine, but it markedly increased the responses of *Mecp2^−/y^* neurons. (**F**) Summary of dopamine-mediated oxidizing responses, in other words, normalized increases in the roGFP ratio (ΔR/Ro), recorded upon modulation of various cellular ROS sources. The dotted horizontal lines refer to the magnitude of the dopamine responses in WT and *Mecp2^−/y^* neurons under control conditions. Asterisks indicate genotype-related differences (* *p* < 0.05, ** *p* < 0.01; unpaired *t*-test). Significant differences as compared to the control response of each genotype (ACSF) are identified by cross-hatches (## *p* < 0.01, ### *p* < 0.001; ANOVA, Holm–Sidak test). The dot plots indicate the scatter of the respective data.

Upon withdrawal of extracellular Ca^2+^ (nominally Ca^2+^-free solutions), noticeable changes in the cytosolic redox baselines were not evident. The subsequent application of dopamine evoked an oxidizing response (Figure 6B), as seen in control solutions (Figure 6A), but its magnitude tended to become reduced only in *Mecp2^−/y^* neurons to 10.0 ± 2.5% (*p* = 0.103, *n* = 4; Figure 6B,F). Mitochondrial uncoupling by FCCP (1 µM) induced a moderate reducing shift in both WT and *Mecp2^−/y^* neurons, indicating that functional mitochondria constantly release small amounts of oxidants into the cytosol. Under these conditions, dopamine elicited unaltered roGFPc responses in WT neurons, whereas a markedly diminished oxidizing response was seen in *Mecp2^−/y^* neurons (5.1 ± 1.3%, *n* = 4; Figure 6C,F). Inhibition of NADPH oxidase by DPI (20 µM) did not evoke noticeable cytosolic baseline changes, but it almost abolished the dopamine-mediated oxidation of roGFPc in both genotypes (Figure 6D,F). Blocking xanthine oxidase by allopurinol (200 µM) did not noticeably affect the baseline redox balance of WT and *Mecp2^−/y^* neurons either. In WT neurons, the dopamine-mediated oxidizing response remained unchanged, but in *Mecp2^−/y^* neurons it was markedly potentiated, now measuring 25.9 ± 9.8% (*n* = 9; Figure 6E,F).

**Figure 7:**
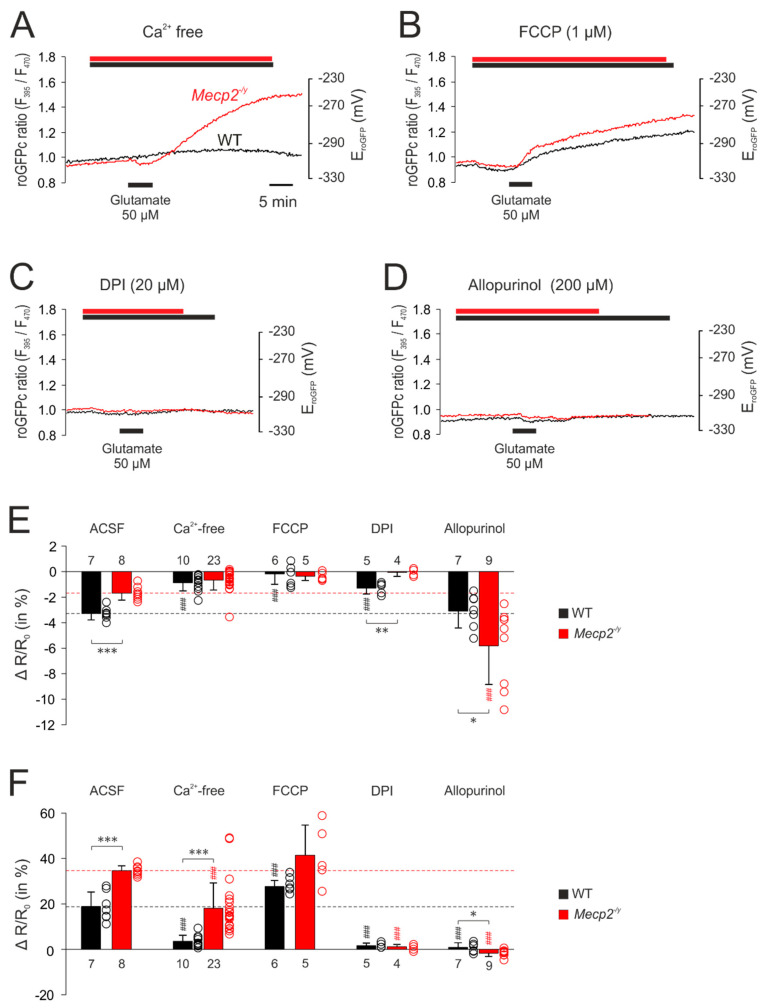
Mitochondrial and extra-mitochondrial sources contribute to the glutamate-mediated oxidation of roGFPc. (**A**) Withdrawal of extracellular Ca^2+^ markedly depressed the glutamate-mediated oxidation of roGFPc in WT neurons and partly reduced it in *Mecp2^−/y^* neurons. (**B**) The uncoupling agent FCCP by itself mediated a slight reduction of roGFPc. Subsequent glutamate application induced a more intense oxidation of roGFPc in WT neurons; in *Mecp2^−/y^* neurons, the glutamate-mediated oxidation tended to increase. (**C**) Blockade of NADPH oxidase by DPI almost abolished the glutamate-mediated oxidizing responses in both genotypes. (**D**) Inhibition of xanthine oxidase by allopurinol severely depressed the glutamate-mediated roGFPc oxidation in both, WT and *Mecp2^−/y^* neurons. (**E**) Modulation of the glutamate-mediated initial reducing shift by the various treatments assessed. Plotted are the normalized changes in the roGFP ratio (ΔR/Ro), and significant differences among genotypes in this and the following panel are indicated by asterisks (* *p* < 0.05, ** *p* < 0.01, *** *p* < 0.001; unpaired *t*-test/Mann–Whitney rank sum test). Significant differences as compared to the control responses of the given genotype (ACSF) are indicated by cross-hatches (### *p* < 0.001; ANOVA, Holm–Sidak test). Dot plots indicate the scatter of the respective data. (**F**) Modulation of the glutamate-induced oxidization of roGFPc. Note that most intense antagonistic effects were observed upon inhibition of NADPH-oxidase and xanthine oxidase.

The corresponding set of treatments was then performed with glutamate-mediated stimulation. Withdrawing extracellular Ca^2+^ almost completely abolished the glutamate-mediated responses in WT neurons. The initial reducing shift was almost absent, and the secondary oxidizing shift was markedly depressed. In *Mecp2^−/y^* neurons, the dampening effects were less pronounced. The initial reducing shift was similar to WT neurons, but the secondary oxidizing response—despite a ~48% depression, was still more intense than in WT neurons (Figure 7A,E,F). Upon mitochondrial uncoupling (1 µM FCCP), glutamate no longer evoked a noticeable initial reducing shift, but immediately elicited a pronounced cytosolic oxidation, which tended to be more intense in *Mecp2^−/y^* (41.4 ± 13.3%, *n* = 5) than in WT neurons (27.7 ± 2.7%; *n* = 6, *p* = 0.082). It also tended to be higher than the cytosolic oxidation observed in control solution (Figure 7B,F). Inhibition of NADPH oxidase by DPI (20 µM) almost completely abolished the responses to glutamate in both genotypes (Figure 7C,F). As shown in the summarizing bar plots, only in WT neurons, part of the initial reducing shift persisted (Figure 7E). Blockade of xanthine oxidase by allopurinol (200 µM) also almost completely abolished the glutamate-mediated oxidizing responses in the cytosol of WT and *Mecp2^−/y^* neurons. Instead, only the initial moderate reducing shift remained, and it became prolonged and was intensified especially in *Mecp2^−/y^* neurons (Figure 7D–F).

## 4. Discussion

In the present study, we characterized the neurotransmitter-mediated redox-changes in mitochondria and the cytosol of cultured hippocampal *Mecp2^−/y^* and WT neurons. Furthermore, by modulatory treatments, we identified the subcellular ROS sources contributing to the respective redox-responses in WT and *Mecp2^−/y^* neurons. An artefactual contribution of extracellular H_2_O_2_ formation arising from autoxidation of the dissolved neurotransmitters (in particular dopamine and norepinephrine [65]) was ruled out.

The cytosolic and the mitochondrial resting redox baselines did not differ among cultured WT and *Mecp2^−/y^* neurons, and a genotype-matched comparison of the two cell compartments did not identify any subcellular differences either (Figure 2C,D). Stimulation of the cultured neurons by neurotransmitters, however, revealed very clear compartment-specific and genotype-related differences. Each of the neurotransmitters provoked more intense oxidizing shifts in cytosol than in mitochondria. Furthermore, each neurotransmitter evoked exaggerated oxidizing responses in the cytosol of *Mecp2^−/y^* neurons as compared to WT neurons. This shows that cytosolic redox-homeostasis is markedly weakened in MeCP2-deficient neurons, as seen earlier in response to anoxia, mitochondrial poisoning and direct oxidant-stress [55,56]. Thus, physiological stimuli such as neurotransmitter signaling are also sufficient to provoke exaggerated oxidizing redox shifts in *Mecp2^−/y^* hippocampus and thereby to challenge proper cell functioning by mediating an oxidative burden. More oxidized cytosolic resting redox-baselines in *Mecp2^−/y^* neurons, as seen earlier in the more mature and structurally more complex organotypic hippocampal slices [55,56], were not evident in the dissociated cell cultures.

In WT neurons, glutamate induced the most pronounced cytosolic oxidation, followed by norepinephrine, dopamine and serotonin. In contrast, the most pronounced cytosolic oxidation of *Mecp2^−/y^* neurons was caused by norepinephrine, followed by glutamate, dopamine and serotonin. In general, dopamine-treated cells recovered best, regaining their redox baselines within minutes. The fact that some neurons did not recover from the other neurotransmitters may arise from either the minute-long treatments, the relatively high transmitter concentrations applied, or both. This was seen in particular with glutamate, which—especially when applied at higher concentrations as the 50 µM used here—may mediate potentially irreversible cell damage via NMDA-receptor activation, massive subsequent Ca^2+^ influx and oxidative stress [78,79]. It should be pointed out though, that this damage occurs on the time scale of several hours or even days. The induction of acute cellular damage in hippocampal cultures requires 10-fold higher glutamate levels (500 µM, 1 min application), which then causes obvious cell swelling, blebbing of dendrites and loss of spines [80]. Yet, in the same hippocampal cultures (DIV 11–18), a 1-min treatment with a slightly lower concentration of 200 µM glutamate was reported to cause only functional disturbances. Despite a near-complete depolarization, the membrane potentials of the hippocampal neurons fully recovered, which was then followed by a long-term depression of evoked excitatory postsynaptic currents for more than 20 min. Just as we observed in our cultured neurons, a sustained cellular Ca^2+^ increase was absent, and a rather rapid (Ca^2+^)_i_ clearance occurred. Any obvious changes in dendritic morphology were not detected in this report [80], which clearly shows that even when applied at higher concentrations, glutamate may also just mediate (transient) functional disturbances.

In mitochondrial matrices, the neurotransmitter-mediated redox-changes were more moderate and more reversible. This may indicate that—despite generating ROS themselves—mitochondria are more efficiently redox-buffered than the cytosolic compartment. Of course it also needs to be considered that a significant fraction of the mitochondria derived ROS is directly produced in the intermembrane space [81], from where it may easily affect the cytosol but not necessarily enter the matrix space. Furthermore, the neurotransmitter-mediated mitochondrial redox responses were more uniform among genotypes. Only serotonin evoked intensified oxidizing shifts in the mitochondrial matrix of *Mecp2^−/y^* neurons.

By pharmacological intervention, withdrawal of extracellular Ca^2+^, as well as ratiometric Fura-2 imaging, we identified the mechanisms contributing to the transmitter-mediated cytosolic redox alterations. Moderate Ca^2+^ rises stimulate mitochondrial metabolism. As the ROS generation of mitochondria correlates with their metabolic rate, this metabolic stimulation may reflect part of the observed cytosolic and mitochondrial oxidizing responses [82]. Excessive cellular Ca^2+^ loads may, however, provoke mitochondrial dysfunction and the production of high amounts of ROS [82,83]. Glutamate mediated the most intense cytosolic Ca^2+^ increases, which—based on time-course and magnitude—represent Ca^2+^-influx from extracellular space. Serotonin, norepinephrine and dopamine evoked markedly less intense cytosolic Ca^2+^ transients by releasing Ca^2+^ from intracellular stores. This clearly heterogeneous nature of the recorded Ca^2+^ transients argues against Ca^2+^ being the sole cause of the transmitter-evoked oxidizing redox responses, which is confirmed by the experiments in Ca^2+^ free solutions.

**Figure 8:**
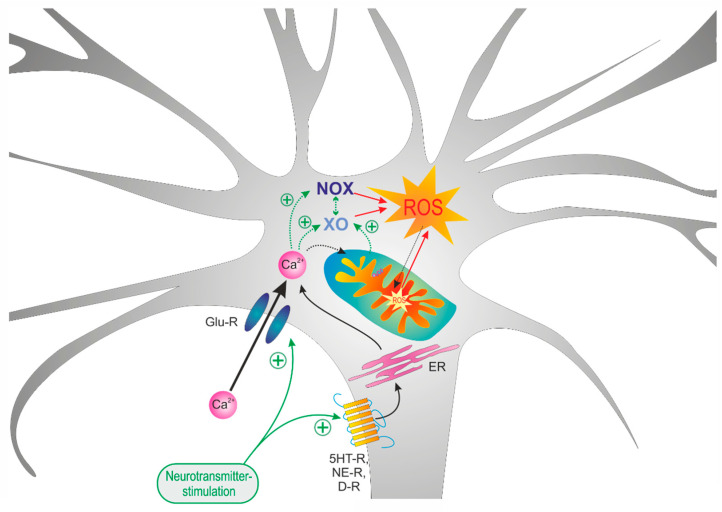
Summarizing schematic showing that mitochondria as well as xanthine oxidase (XO) and NAHPH oxidase (NOX) differently contribute to the neurotransmitter-mediated oxidizing responses. In the case of glutamate, massive Ca^2+^ influx mediates a downstream activation of XO and NOX. In contrast, in the case of dopamine stimulation, which only mediates moderate Ca^2+^ transients, mitochondria as well as NOX were identified as the main mediators of the oxidizing cellular responses.

Ca^2+^ withdrawal efficiently reduced the cytosolic oxidation induced by glutamate in both genotypes, but the genotypic differences persisted. This identifies Ca^2+^ influx as one crucial component of the redox-alterations in both genotypes, at least for stimulation by glutamate (Figure 8). The dopamine-induced oxidation, was however, diminished upon Ca^2+^ withdrawal in *Mecp2^−/y^* neurons only, which now showed response levels just as with WT neurons. Various reports confirm disturbed intracellular Ca^2+^ signaling, buffering and homeostasis in *Mecp2^−/y^* neurons and glial cells [84,85,86,87]. The only moderate Ca^2+^ transients induced by dopamine did, however, not differ among *Mecp2^−/y^* and WT neurons. Hence, the exaggerated dopamine-mediated redox-responses in *Mecp2^−/y^* neurons cannot arise from a cellular Ca^2+^-overload itself. They rather involve ROS-generating events downstream of Ca^2+^-signaling, as in Ca^2+^ free solutions, the genotypic differences in the redox responses to dopamine were abolished.

This is where mitochondria, which are considered a crucial factor in the redox-imbalance in RTT, come into play. An increased O_2_ consumption and H_2_O_2_ release were confirmed earlier for mitochondria isolated from hippocampus and cortex of symptomatic (7 week old) *Mecp2^−/y^* mice [55] or from the full brains of symptomatic male [54] and female *Mecp2*-mutant mice [59]. Our most recent report confirms that the exaggerated H_2_O_2_ release by mitochondria represents a life-long burden which affects all brain regions of male as well as female MeCP2-deficient mice [72]. Therefore, we assessed the contribution of mitochondrial ROS generation to the neurotransmitter-mediated oxidizing responses by chemical uncoupling, following the concept that mitochondrial ROS generation depends on a sufficient mitochondrial membrane potential [88,89].

FCCP evoked a noticeable reducing shift in the roGFPc ratio, indicating that mitochondrial ROS release constantly challenges cytosolic redox homeostasis. In the further experiments, FCCP markedly diminished the dopamine-mediated oxidation in *Mecp2^−/y^* neurons only, thereby reversing the genotypic differences. This confirms that in *Mecp2^−/y^* neurons, mitochondria—possibly stimulated by the moderate (Ca^2+^)_i_ rise [82]—markedly contribute to the dopamine-mediated cytosolic oxidation. In contrast, the glutamate-mediated oxidation in WT neurons increased upon FCCP treatment and it tended to be higher in *Mecp2^−/y^* neurons. This shows that during glutamate-mediated stimulation, mitochondria are protective, possibly by buffering part of the pronounced cellular Ca^2+^ load and by providing chemical energy for the re-instatement of normal ion distribution (Figure 8). Furthermore, all of the above also indicates that non-mitochondrial cell-endogenous ROS sources contribute to the activity-related subcellular redox changes as well.

Indeed, by far the most pronounced dampening effects occurred with DPI, an inhibitor of NOX, which almost completely blocked the oxidizing responses to dopamine and to glutamate in both genotypes and thereby abolished all genotype-related differences. This identifies the cytosolic ROS production by NOX as the key factor for the redox-alterations during altered neuronal activity. In hippocampal neurons, an active NADPH oxidase was confirmed to not only contribute to intracellular ROS production [90], but to be the main source of NMDA-receptor mediated ROS generation [91]. In anoxia/reoxygenation, NOX seems to be activated by a (Ca^2+^)_i_ increase [92]. A similar Ca^2+^-dependence is suggested by the markedly depressed cytosolic oxidation in WT neurons observed in Ca^2+^-free solutions upon glutamate stimulation. Accordingly, it is NOX, which plays a key role in the transmitter-mediated oxidizing responses in WT and *Mecp2^−/y^* neurons (Figure 8). This nicely correlates with findings in Rett-patient fibroblasts, in which an aberrant constitutive NOX-activation was unveiled as a crucial factor for the cellular redox-imbalance and the oxidative stress [58].

Similar to what was seen with DPI, allopurinol, an inhibitor of XO, also almost abolished the glutamate-mediated oxidation of WT and *Mecp2^−/y^* neurons. As either drug efficiently blocked the glutamate effects; this identifies NOX as well as XO as central players in the cell endogenous cytosolic ROS production. Furthermore, an interaction or sequential activation of both oxidases has to be assumed (Figure 8). Such interactions have been unveiled in pheochromocytoma PC12 cells, in which intermittent hypoxia activates XO, which—by elevating (Ca^2+^)_i_—stimulates the NAPDH oxidase to generate ROS [93]. Furthermore, a strong cellular Ca^2+^ load via activation of calpain may irreversibly convert XDH into the superoxide producing XO [94,95]. Likewise, mitochondria under the conditions of stress were proposed to release a protease factor that irreversibly converts XDH into XO [82,96].

The dopamine-responses in WT neurons were, however, not affected by allopurinol, and the oxidizing transients in *Mecp2^−/y^* neurons even increased under these conditions. Thus, the cooperativity of XO and NOX does not apply to the more moderate cytosolic redox- and Ca^2+^-responses elicited by dopamine. For this neurotransmitter rather mitochondria and NOX appear as the pivotal mediators underlying the observed cytosolic redox shift.

In conclusion, physiological stimuli such as alterations in neuronal activity mediated by neurotransmitter signaling are capable of inducing overshooting redox-alterations in MeCP2-deficient neurons. Various neurotransmitter receptors and ion-channels are among the potentially redox-regulated proteins [97], and increased levels of oxidants have been confirmed to modulate neuronal excitability and to depress synaptic plasticity [98,99]. In view of the markedly impaired neurotransmitter signaling in RTT and the altered excitatory/inhibitory balance [40,42], these overshooting redox-responses may present a further challenge for neuronal function. In particular, as these exaggerated redox responses are already evident in neonatal neurons. Here, only hippocampal neurons were studied and the question remains to be solved, to what extent such overshooting redox-responses are also present in other brain regions. Furthermore, our study was conducted on male mice only, to ensure a total lack of MeCP2 in each cell studied. In female, heterozygous *Mecp2*-mutant mice, this would of course apply to only those cells transcribing the mutant *Mecp2* allele. Hence, due to the X chromosome mosaicism, on the neuronal-network level each cell might not be directly affected by the neurotransmitter-mediated redox distortions. Nevertheless, the RTT-related oxidative stress, the synaptic alterations and the network dysfunctions have been confirmed to also apply to the heterozygous condition, albeit at a more heterogeneous and often less severe manifestation than in hemizygous males [59,72,100,101]. Therefore, the neuronal activity-related exaggerated redox responses unveiled here have to be considered as clearly relevant for the disturbed network function in RTT, as they constitute a crucial connecting point, where altered neuronal signaling and impaired subcellular redox-homeostasis do converge in the course of RTT pathogenesis.

## Abbreviations

ΔR/R_0_	change in fluorescence ratio, normalized to control ratio
ACSF	artificial cerebrospinal fluid
DIV	days in vitro
DMSO	dimethyl sulfoxide
DTT	1,4-dithio-DL-threitol
DPI	diphenyleneiodonium chloride
E^0′^_roGFP_	standard redox potential
E_roGFP_	roGFP reduction potential
F 380_max/_F 380_min_	fluorescence ratio at 380 nm excitation, Ca^2+^-free/Ca^2+^-saturated
F 395/F 470	fluorescence ratio at 395 nm excitation and 470 nm excitation
F 470_ox_	fluorescence intensity at 470 nm excitation, fully oxidized
F 470_ox/_F 470_red_	fluorescence ratio at 470 nm, fully oxidized/fully reduced
F 470_red_	fluorescence intensity at 470 nm excitation, fully reduced
FCCP	carbonyl cyanide 4-(trifluoromethoxy)phenylhydrazone
FCS	fetal calf serum
HBSS	Hanks’-balanced salt solution
HEPES	4-(2-hydroxyethyl)piperazine-1-ethanesulfonic acid
MAP2	microtubule associated protein 2
MeCP2	methyl-CpG binding protein 2, protein
*MECP2*	methyl-CpG binding protein 2, encoding gene (human)
*Mecp2*	methyl-CpG binding protein 2, encoding gene (mouse)
*Mecp2^−/y^*	MeCP2-deficient male mouse (hemizygous)
*Mecp2^+/−^*	MeCP2-deficient female mouse (heterozygous)
MEM	minimal essential medium
NOX	NADPH oxidase
OxD_roGFP_	relative degree of roGFP oxidation
PBS	phosphate buffered saline
R	fluorescence ratio
roGFP	reduction oxidation-sensitive green fluorescent protein
roGFPc	roGFP expressed in cytosol
roGFPm	roGFP expressed in mitochondrial matrix
ROS	reactive oxygen species
R_ox_	ratio corresponding to full oxidation
R_red_	ratio corresponding to full reduction
RTT	Rett syndrome
SOD1	superoxide dismutase 1, Cu/Zn superoxide dismutase
WT	wildtype
XO	xanthine oxidase
XDH	xanthine dehydrogenase
